# Differences in pathogenicity of three animal isolates of *Mycobacterium* species in a mouse model

**DOI:** 10.1371/journal.pone.0183666

**Published:** 2017-08-24

**Authors:** Haodi Dong, Yue Lv, Srinand Sreevatsan, Deming Zhao, Xiangmei Zhou

**Affiliations:** 1 State Key Laboratory of Agrobiotechnology, Key Laboratory of Animal Epidemiology of the Ministry of Agriculture and College of Veterinary Medicine, China Agricultural University, Beijing, China; 2 Veterinary Population Medicine Department, College of Veterinary Medicine, University of Minnesota, St Paul, MN, United States of America; Rutgers Biomedical and Health Sciences, UNITED STATES

## Abstract

Animal mycobacterioses are among the most important zoonoses worldwide. These are generally caused by either *Mycobacterium tuberculosis* (MTB), *M*. *bovis* (MBO) or *M*. *avium* (MAV). To test the hypothesis that different species of pathogenic mycobacteria isolated from varied anatomic locations or animal species differ in virulence and pathogenicity, we performed experiments with three mycobacteria strains (NTSE-3(MTB), NTSE-4(MBO) and NTSE-5 (MAV)) obtained from animal species. Spoligotyping analysis was used to confirm both MTB and MBO strains while the MAV strain was confirmed by 16s rDNA sequencing. BALB/c mice were intranasally infected with the three strains at low and high CFU doses to evaluate variations in pathogenicity. Clinical and pathological parameters were assessed. Infected mice were euthanized at 80 days post-inoculation (dpi). Measures of lung and body weights indicated that the MBO infected group had higher mortality, more weight loss, higher bacterial burden and more severe lesions in lungs than the other two groups. Cytokine profiles showed higher levels of TNF-α for MBO versus MTB, while MAV had the highest amounts of IFN-β *in vitro* and *in vivo*. *In vitro* levels of other cytokines such as IL-1β, IL-10, IL-12, IL-17, and IFN-β showed that Th1 cells had the strongest response in MBO infected mice and that Th2 cells were inhibited. We found that the level of virulence among the three isolates decreased in the following order MBO>MTB>MAV.

## Introduction

Tuberculosis (TB) caused by *Mycobacterium tuberculosis* and *Mycobacterium bovis* remains as a serious zoonosis and is responsible for 2 million annual deaths worldwide. Current estimations indicate that one third of the world’s population is infected with *M*. *tuberculosis*, with 9 million people becoming sick each year with TB [1]. Globally, the incidence of TB is highest in Africa, South Asia and West Pacific [2]. To date, the protective effects of the only recognized vaccine, Bacillus Calmette-Guerin (BCG), for both humans and animals, varies largely, and these variations are probably associated with the differences in virulence of different bacterial strains [3].

Tuberculosis is distributed widely in wild animals, especially in some developed countries, such as deer in the United States, European wild boar in Spain, badgers in the UK and brush-tailed possums in New Zealand [4].Thus, wildlife has been considered as a reservoir in the maintenance of tuberculosis infection in those regions of the globe [4].The distribution of tuberculosis in wildlife may also have severe consequences for livestock due to spillover epidemics at wildlife-livestock interface. This situation is worse in some developing countries [4], where there are multifaceted impacts on animal (domesticated and wildlife) as well as human health. Wide distribution of tuberculosis in wildlife and domestic animals cause great difficulties for the control and prevention of tuberculosis.

The pathology of tuberculosis infection has been well characterized and the main transmission route has been verified as inhalation of contaminated aerosols. However, *Mycobacterium* strains isolated from diverse animal species at different times and geographic locations seem to show a variety of pathogenicities. In this study, we tested three M*ycobacterium* species—one isolated from beef steer, one from a dairy cow and a third from a deer. The three strains were administered intranasally to mice at identical CFU doses. Each strain showed different disease severity in terms of clinical manifestation, lung pathology and tissue bacterial burden, which indicates diversity in the pathogenicity among M*ycobacterium* strains. Moreover, cytokines were evaluated by ELISA to observe differences in the immune reponses elicited by different *Mycobacterium species*.

## Materials & methods

### Ethics statement

All animal experiments were performed according to the Chinese Regulations of Laboratory Animals—The Guidelines for the Care of Laboratory Animals (Ministry of Science and Technology of People's Republic of China) and Laboratory Animal Requirements of Environment and Housing Facilities (GB 14925–2010, National Laboratory Animal Standardization Technical Committee).

The animal studies and research protocols were approved by The Laboratory Animal Ethical Committee of China Agricultural University under the license number- 20110611–0.

### Animals

Eight-week-old female SPF BALB/c mice were used in this study. Mice were acclimatized for 10 days before experiments. They were housed in the rodent cabinets in a Biosafety Level 3 Laboratory in China Agricultural University with food and water provided ad libitum.

### Bacteria

The three species of *Mycobacterium* used in this study were obtained from the China Institute of Veterinary Drug Control. These included strains NTSE-3 (*M*. *tuberculosis*—MTB), NTSE-4 (*M*. *bovis*—MBO) and NTSE-5 (*M*. *avium—*MAV). NTSE-3 was isolated from a beef cattle with tuberculosis in Taiwan; NTSE-4 was isolated from a dairy cow in Beijing; and NTSE-5 was isolated in Tianjin, from a deer with lesions that were similar to those seen in bovine TB. All strains were grown in Middlebrook 7H9 broth (BD Biosciences, NJ,USA) supplemented with 0.2% (v/v) glycerol, 10% oleic albumin dextrose catalase enrichment (Difco) and 0.02% (v/v) Tween-80 at 37°C [5]. All bacteria used in the experiments were in mid log-phase and resuspended in PBS.

### Experimental mouse model of pulmonary TB

Mice (n = 5 per group) were anesthetized by Zoletil 50 (Virbac, France), which was first diluted 5-fold and 50-μl (0.5mg) was injected intraperitoneally per mouse. Experimental mice were separated into 4 dosage groups (1×10^5^, 1×10^4^, 2.5×10^3^ and 1×10^3^ CFU of bacteria, respectively), each group containing 5 mice. The animal challenges were conducted via nasal inhalation according to the protocol described elsewhere [6]. Control group mice received intranasal PBS. All mice were maintained in the cages fitted with micro-isolators. Mice’s health was controlled daily checking for smoothness of the fur, movement and activity, and reaction to outside stimuli. Mice were weighed every week and sacrificed when their weight was 25% lower than the average weight of control group [7]. The remaining mice were euthanized at 80 days post-infection and necropsied.

### IFN-β and TNF-α measurement in serum from infected mice

Blood was collected from the orbits of infected mice. Serum was harvested by centrifugation of clotted blood for 15min at 1000×g. Serum was aspirated from the top and frozen until used. IFN-β and TNF-α cytokine levels were analyzed by Colorimetric Sandwich ELISA (Proteintech, Wuhan, China) according to the manufacturer’s instructions.

### Cytokine measurement in mouse macrophage cell line

The RAW264.7 cell line was obtained from the National Infrastructure of Cell Line Resources and cultured in DMEM (Hyclone, Logan, UT, USA) with 10% FBS (Gibco, Grand Island, NY, USA). RAW264.7 cells were infected with 10 MOI of the three strains of *Mycobacterium*. Cell culture supernatants were collected at 6h, 12h, 24h post infection and cytokine levels were measured by Colorimetric Sandwich ELISA (Proteintech, Wuhan, China) according to the manufacturer’s instructions.

### Preparation of tissue for morphological observation

All animals were weighed prior to euthanasia. Euthanasia was performed by cervical dislocation and then mice were submerged into 75% ethyl alcohol between 1 and 2 min. Lungs and spleens of the mice were aseptically removed. The weight of each lung was calculated and the gross anatomic picture was taken in order to document the extent of the lesions.

### Histological observation

The right lungs were removed and steeped in formalin for 7 days, and then dehydrated and embedded in paraffin using routine protocols. Tissues were sectioned at 2μm, stained with hematoxylin and eosin and Ziehl-Neelsen, and subsequently mounted with balsam (Leica CV Ulrea).

### Determination of CFU in lungs and spleens

The left lung and spleen of each mouse were ground with 1ml sterile PBS in a sterile burnisher. 50μl of each homogenate were spread onto 4 duplicate plates containing Middlebrook 7H11 (BD Biosciences, NJ, USA) supplemented with Tween 80, glycerol, pyruvate, catalase, bovine serum albumin (BSA) and dextrose-enriched medium. The plates were incubated at 37°C with 5% CO_2_. The number of colonies was counted 4-weeks post-incubation.

### DNA extraction and 16S rDNA sequencing

Nucleic acids of mycobacteria were extracted from tissues using a commercial RTP Mycobacteria Kit (Stratec molecular, Berlin, Germany), according to the manufacturer’s instructions. Each strain of bacteria contained at least four colonies collected from fresh 7H9 plates. 16S rDNA was sequenced by Sangon Biotech (Shanghai, China). Spoligotyping was performed to confirm species of the two *Mycobacterium tuberculosis Complex* isolates.

### Statistical analysis

Statistical analysis was performed using one-way ANOVA with GraphPad Prism 5 (GraphPad, CA, USA). P≤0.05 was considered statistically significant. Results are expressed as mean ± SEM. The P values shown in the figures are presented with *** (p<0.001), ** (p <0.01), *(p<0.05) or NS (not significant).

## Results

Three strains of M*ycobacterium* were used into this study to evaluate the variations in infectivity in the experimental mouse model. Sequencing analysis of the 16S ribosomal DNA (16S rDNA) [8] revealed that strains NTSE-3 and NTSE-4 were *Mycobacterium tuberculosis* Complex (MTBC) with 99% and 98% nucleotide similarities, respectively, while strain NTSE-5 was *Mycobacterium avium* (MAV) with 96% nucleotide similarities. *Mycobacterium tuberculosis* Complex isolates (NTSE-3 and NTSE-4) were further analyzed by spoligotyping to confirm species and genotype (S1 Fig).

### Differences in the severity of the clinical manifestation of infection among mice exposed to different mycobacteria strains

**Fig 1:**
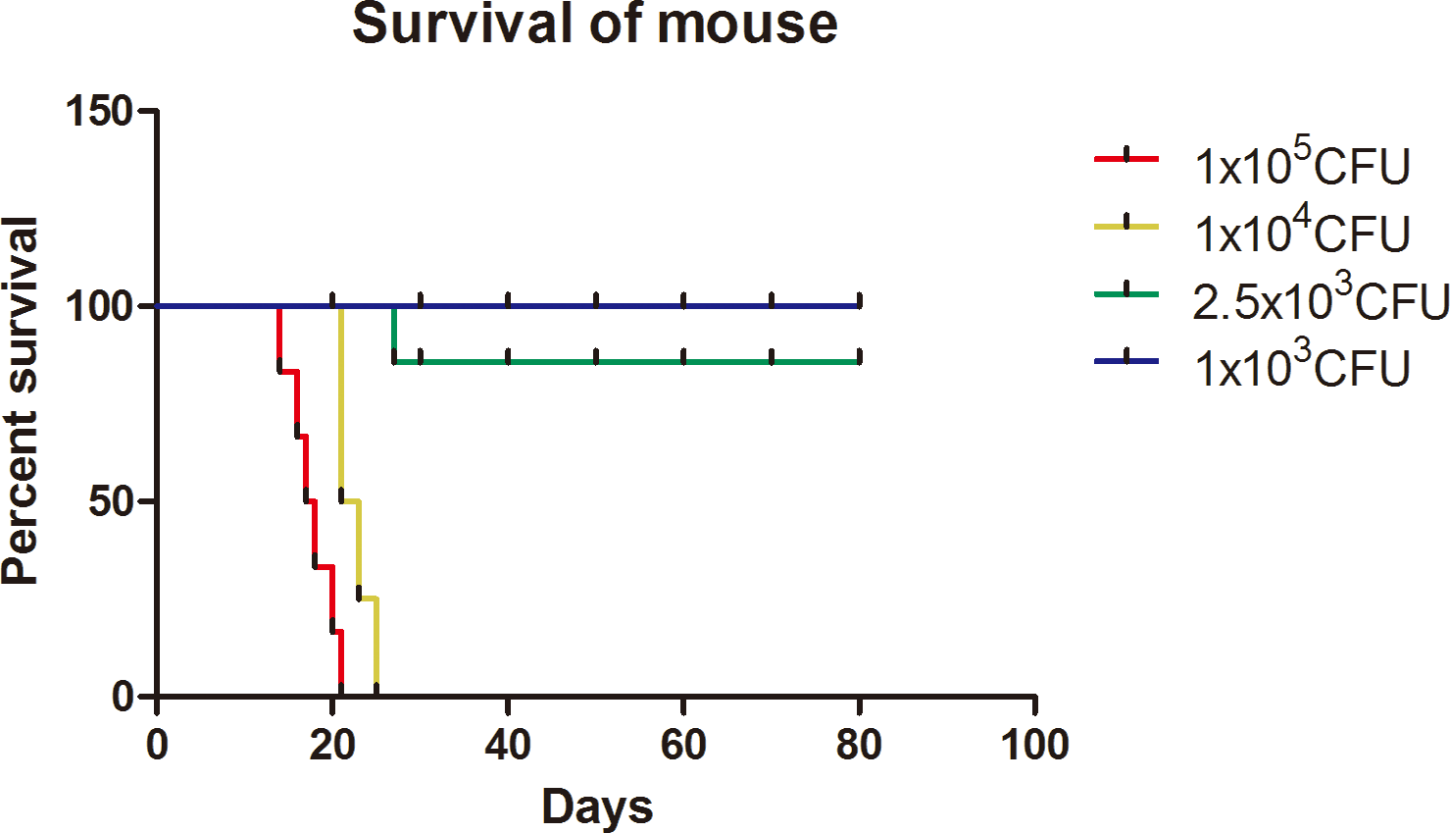
Survival of mice infected with the NTSE-4 strain. Mice were infected with four different dosages of the MBO strain through intranasal inhalation. Each dosage group had six mice. Observation times (day post-inoculation) are indicated in the X-axis.

Mice were inoculated with different dosages of the three bacterial strains via nasal inhalation and the clinical observations persisted 80 dpi. A similar procedure was carried out for the mice control group, inoculated with PBS. During the observation period of 80 days, clinical symptoms such as irregular hair coat and poor behavioral attitude were found as infection progressed. Clinical signs at 80 dpi are shown in Table 1. Mice challenged with MTB and *M*.*avium* strains were healthy until the end of observation (80 dpi), regardless of the administered dosages of bacteria. In contrast, all five mice infected with higher dosages of MBO strains died between 17 dpi (1×10^5^ CFU) and 22 dpi (1×10^4^ CFU), with a median of 19 and 23 days, respectively (Fig 1). One mouse in the group of 2.5×10^3^ CFU dose died on 28 dpi and the remaining four survived the remainder of the study period, while all the five mice in the lowest dosage group (1×10^3^ CFU) survived for 80 days (Fig 1). Clearly, under our experimental conditions, the three tested *mycobacteria* demonstrated different virulence patterns on the experimental rodents, with MBO being the most lethal.

**Table 1:** 

	Irregular hair coat	Poor spirits	Poor reaction to outside stimulation
**MTB**			
**1x10**^**5**^ **CFU**	5/5	5/5	5/5
**1x10**^**4**^ **CFU**	4/5	4/5	3/5
**2.5x10**^**3**^ **CFU**	1/5	2/5	1/5
**1x10**^**3**^ **CFU**	0/5	1/5	0/5
**MBO**			
**1x10**^**5**^ **CFU**	N/A	N/A	N/A
**1x10**^**4**^ **CFU**	N/A	N/A	N/A
**2.5x10**^**3**^ **CFU**	4/4	4/4	3/4
**1x10**^**3**^ **CFU**	5/5	4/5	3/5
**MAV**			
**1x10**^**5**^ **CFU**	5/5	5/5	5/5
**1x10**^**4**^ **CFU**	4/5	3/5	3/5
**2.5x10**^**3**^ **CFU**	1/5	1/5	0/5
**1x10**^**3**^ **CFU**	0/5	0/5	0/5

**Fig 2:**
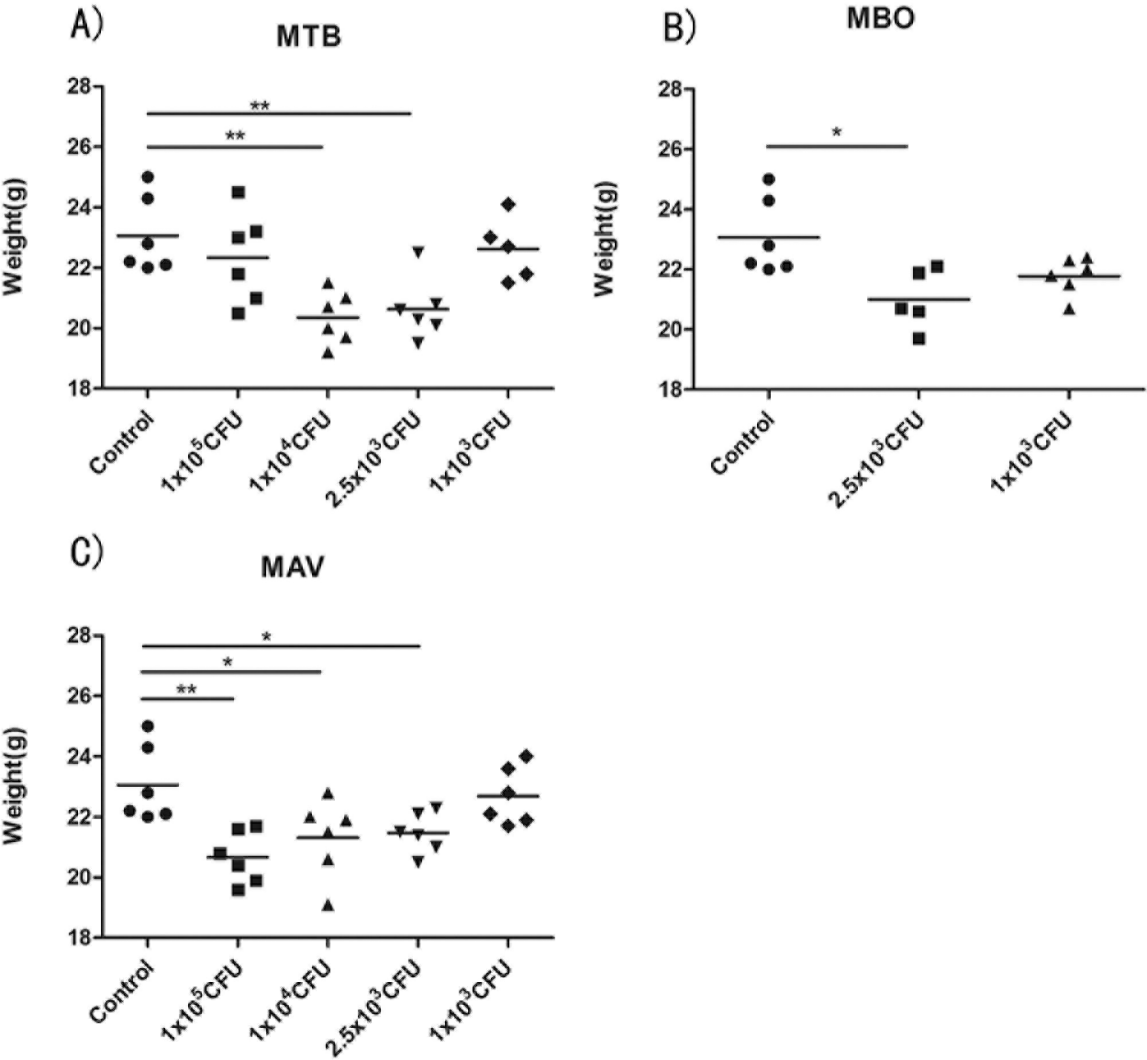
**Change in weight of mice infected with different concentrations of MTB (A), MBO (B) or MAV (C).** Each group had six mice and the experiments were repeated three times independently. Statistical analysis was performed using one-way ANOVA. ***P<0.0001 were considered significantly different.

We next checked the weight of mice infected with different strains after euthanasia at 80 dpi. In the lowest dosage groups (1×10^3^ CFU), mice weight for all the three strains did not show significant differences compared with mice from the control group (Fig 2). For MBO, the weight of mice of the two highest dosage groups (1×10^5^ CFU and 1×10^4^ CFU) were low (12.7g and 11.8g, respectively). Data is not shown because they died before the end of the study duration (Fig 2B). The weights of MTB and MAV groups shows significantly decrease when compared to controls in the high concentration groups (Fig 2A and 2C).However, for the 1x10^5^ CFU group of MTB, the increase in lung weight -due to inflammation and granuloma- was bigger than the decrease in body weight, when compared with the lower dosage group. So the weights of the 1x10^5^ CFU group were bigger than the weights of mice infected with lower dosages.

### Different severities in lung pathology in mice infected with different mycobacteria strains

**Fig 3:**
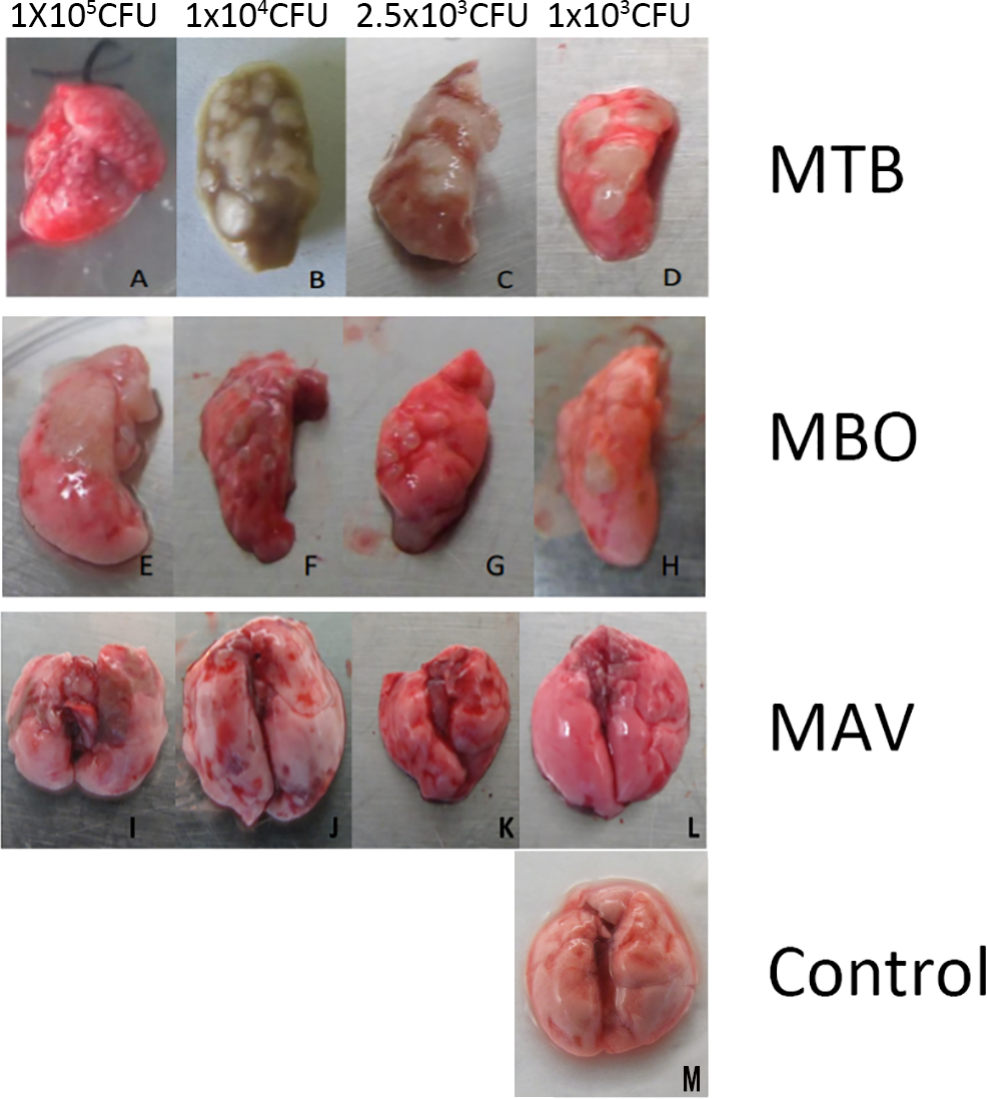
Representative anatomic morphology of the lungs from mice infected with different strains of *Mycobacterium*. The lungs were aseptically removed from the mice either at the end of the clinical observation (80 dpi) or at 18 dpi before death. Pictures of the lungs of mice infected with strains MBO (panel A to D), MTB (panel E to H) and MAV (panel I to L) are shown individually. M shows the lungs of the mice inoculated with PBS. The dosages of inoculums are indicated above.

Lungs from normal mice presented pink, smooth surfaces without detectable bleeding or lesions. In contrast, lungs from infected mice showed clear strain and dose-dependent abnormalities. As shown in Fig 3, large amounts of tubercle and granulomas were observed in the lungs from mice infected with *M*. *bovis* (A to D). This was also seen in lungs from mice from the groups that received lower infection dosages (2.5×10^3^ and 1×10^3^ CFU) of *M*. *bovis*. Mice infected with MTB (E to H) and MAV (I to L) showed markedly milder abnormalities. Lung lesions were detected only in mice inoculated with the higher concentrations of bacteria (1×10^5^and 1×10^4^ CFU of MTB; 1×10^5^ CFU of MAV).

**Fig 4:**
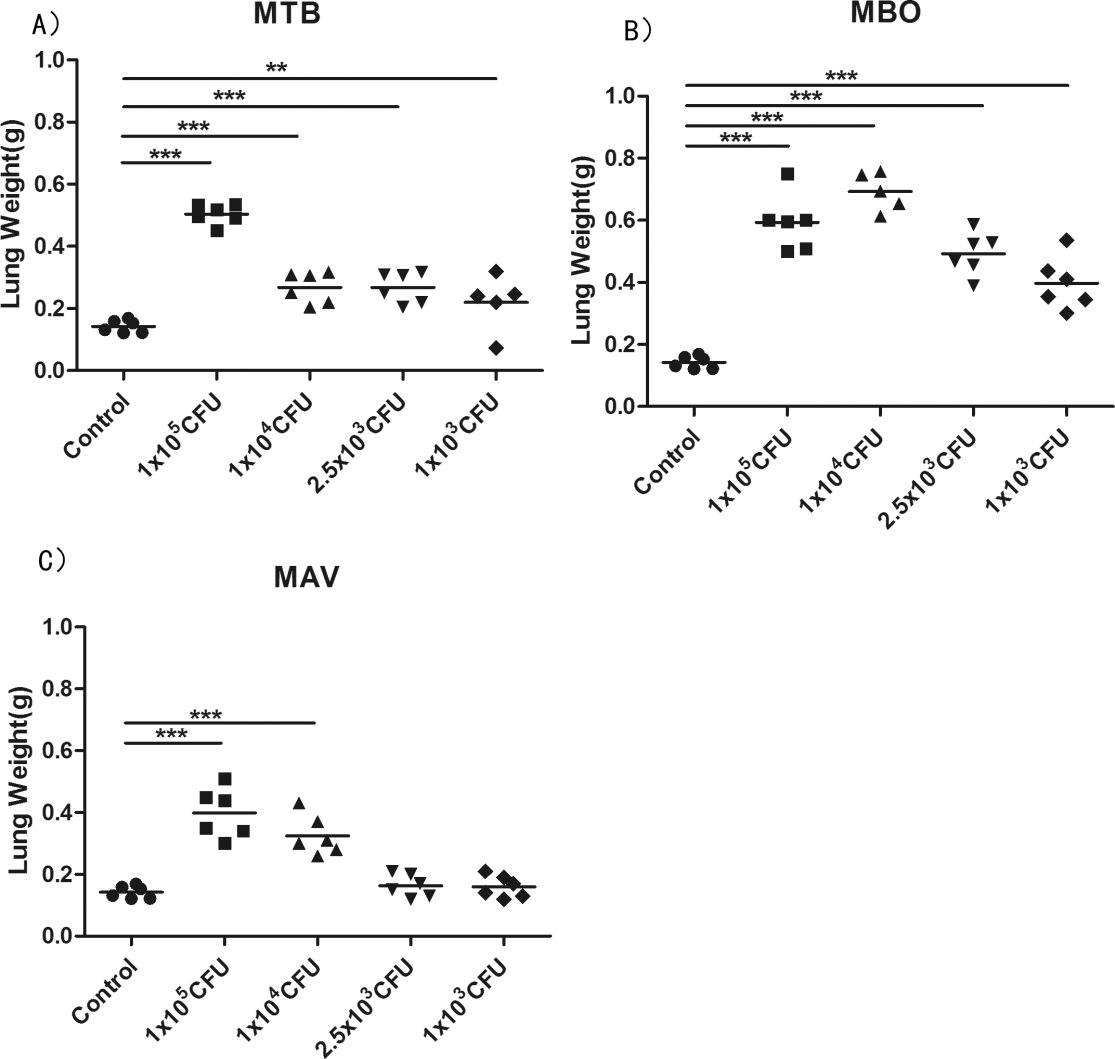
**Comparison of lungs weights from mice infected with different dosages of strains MTB (A), MBO (B), MAV (C).** Each group had six mice. The median weight for each group is indicated. Statistical analysis was performed using one-way ANOVA. ***P<0.0001 were considered significantly different.

The lung weights of each individual mouse, collected either at clinical termination or at the end of the observation period (80 dpi), were measured. As expected, the lung weights of the infected mice were higher than those of normal controls. In the group of the MBO strain, the average lung weights increased more than 3-fold in the mice infected with high dosages of bacteria and roughly 2-fold in those with low dosages, showing significant differences (Fig 4B). Mice infected with high dosages of the MTB strain (1×10^5^, 1×10^4^ and 2.5×10^3^ CFU Fig 4A) or the MAV strain (1×10^5^ and 1×10^4^ CFU, Fig 4C) also revealed significantly heavier lungs than the control group. This suggests severe inflammation and swelling in the lungs among the infected mice.

**Fig 5:**
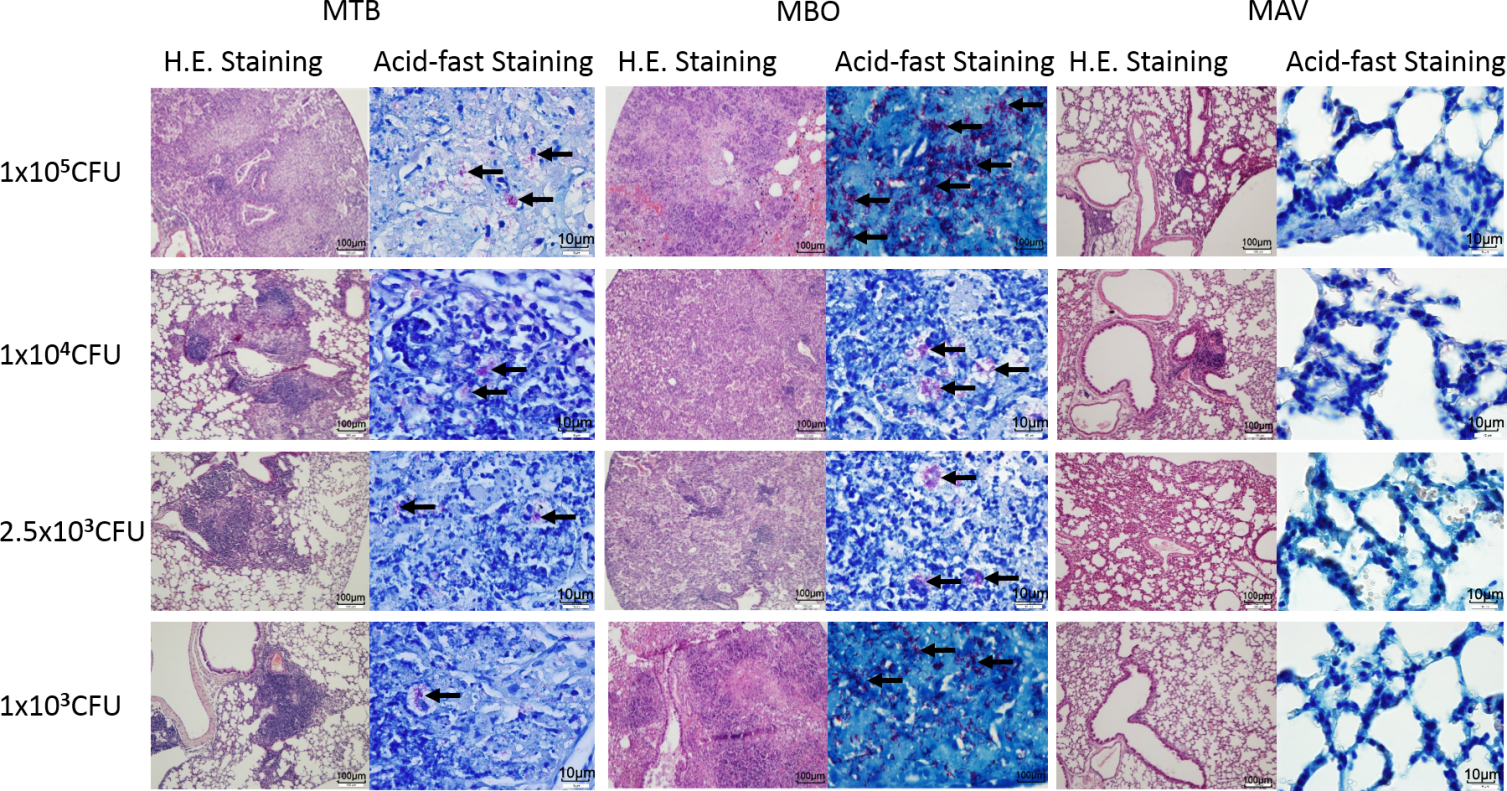
Representative images of lung sections of various infected mice in H&E staining (10X magnification) and acid-fast staining (100X magnification). Various dosages of inoculums are indicated on the left and bacteria strains are shown above.

We used lung sections from infected mice for histological observations. Consistent with clinical observations and anatomic lung abnormalities, we observed neutrophil and monocyte infiltration and large areas of necrosis in the tissues of all mice infected with the MBO strain, as well as in mice infected with high dosages of both MTB and MAV strains (Fig 5). Acid-fast staining revealed red-colored mycobacterium-like structures in the lung sections of mice infected with MBO and MTB strains, as well as in the mice infected with high dosages of MAV (Fig 5). Comparing the three infected groups, mice infected with the MBO strain showed remarkably more severe histopathological changes and increased numbers of acid-fast staining bacilli in the lung tissues, followed by those infected with the MTB strain, while mice infected with the MAV strain showed mild histopathological abnormalities and significantly fewer mycobacteria in the lung tissues (Fig 5).

### Differences in bacterial burdens in lungs and spleens of mice infected with different *Mycobacterium* species

**Fig 6:**
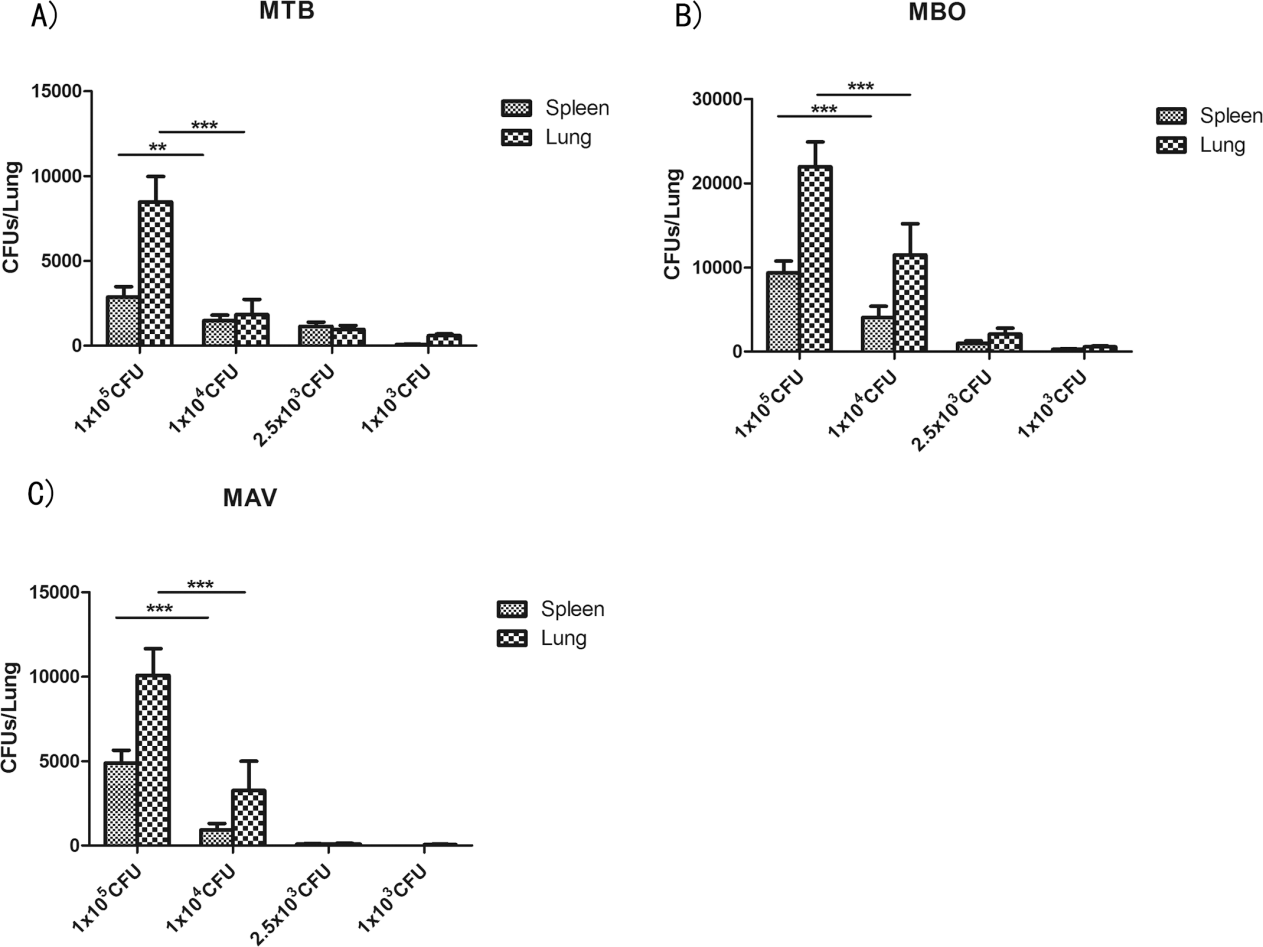
**CFU counting in lungs and spleens from mice infected with MTB (A), MBO (B) and MAV(C).** Tissue homogenates of each individual mouse were cultured in four plates and CFUs were counted and averaged. The CFU value of each group was averaged with the individual data from the six mice of the group and shown as mean±SD. Different dosages of inoculums are indicated at the bottom. Statistical analysis was performed using one-way ANOVA. ***P<0.0001 were considered significantly different.

Lung and spleen homogenates of the infected groups were cultured for mycobacterium. Four weeks post-incubation, CFUs were counted. CFUs in the lung tissues were markedly higher than those of spleen for all three infection groups, highlighting higher bacterial burdens in lungs (Fig 6). In addition, CFUs in lung and spleen tissues of mice infected with the MBO strain were significantly higher than those of the MTB and MAV strains (Fig 6). In the preparations of 1×10^5^ CFU, average CFUs of the MBO infection group were 21,951 in lung and 9,352 in spleen, while CFUs of the MTB and MAV strains were 8,466 and 10,083 in lung and 3,211 and 4,866 in spleen, respectively. The numbers of CFUs decreased with decreasing inoculating dosages of mycobacterium, but were still detectable even in the lowest dosage groups.

### Cytokine production in mice infected with different mycobacterial species

**Fig 7:**
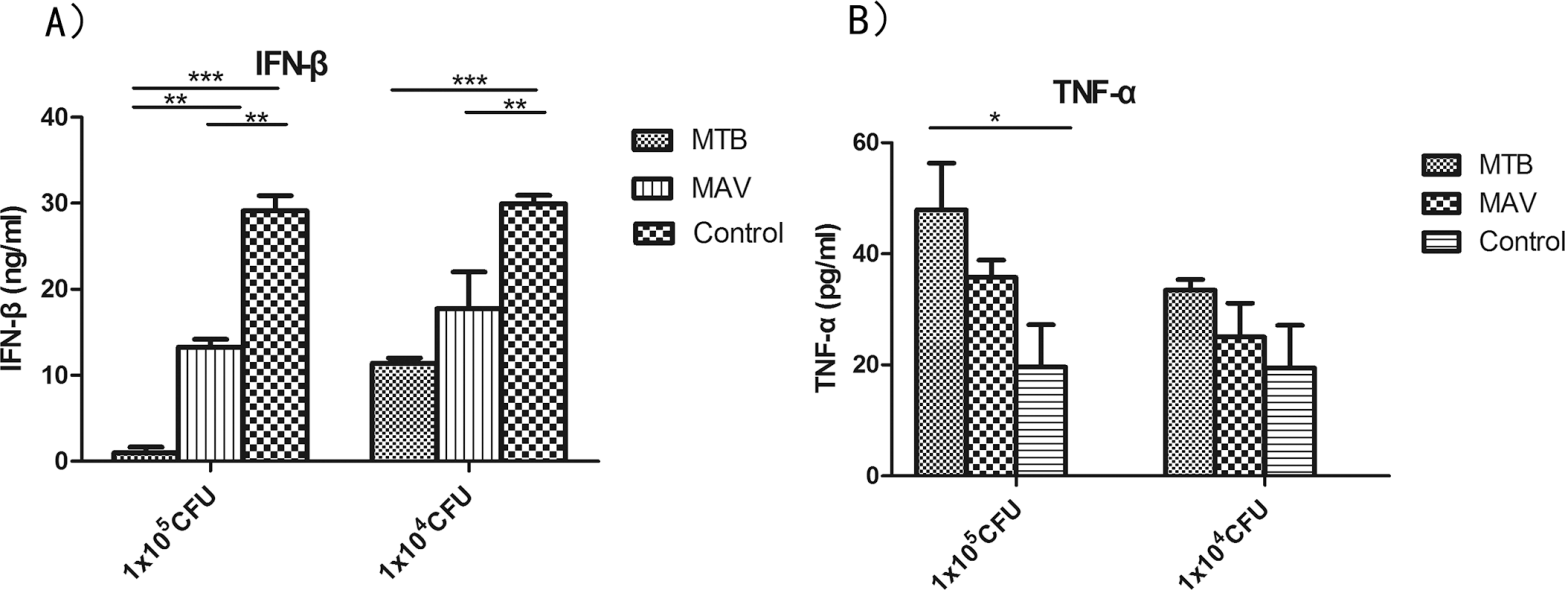
**Expression levels of IFN-β (A) and TNF-α (B) in serum from the high dosage group (1x10**^**5**^ **CFU, 1x10**^**4**^ **CFU) of mice infected with MTB and MAV isolates was detected by ELISA.** Results from each group was averaged with the individual data from the six mice of the group and shown as mean±SD. Statistical analysis was performed using one-way ANOVA. ***P<0.0001 were considered significantly different.

To determine whether immune responses differed by infecting strain and dose, we measured the IFN-β and TNF-α levels in serum from infected and control mice (Fig 7). The MBO group is not shown because all mice died before or at 22 dpi with high dosage of the MBO strain. IFN-β levels were lower in the 1x10^5^ CFU dosage group than in the 1x10^4^ CFU dosage group and were inhibited after infection with MTB, compared to the control group (Fig 7A). In each dosage group, IFN-β levels of MTB infected mice were significantly lower compared to those in the control group, while IFN-β levels of MAV infected mice were lower than those in the control group but not statistically significant. On average, TNF-α level was higher in the 1x10^5^ CFU dosage group than in the 1x10^4^ CFU dosage group. All infected mice produced more TNF-α than mice in the control groups and the TNF-α levels in MTB infected mice were significantly higher than those in mice in the control group (Fig 7B). This suggests that the MTB strain triggered a stronger pro-inflammatory response than the MAV strain.

### Cytokine expression after infection with different mycobacterium species *in vitro*

**Fig 8:**
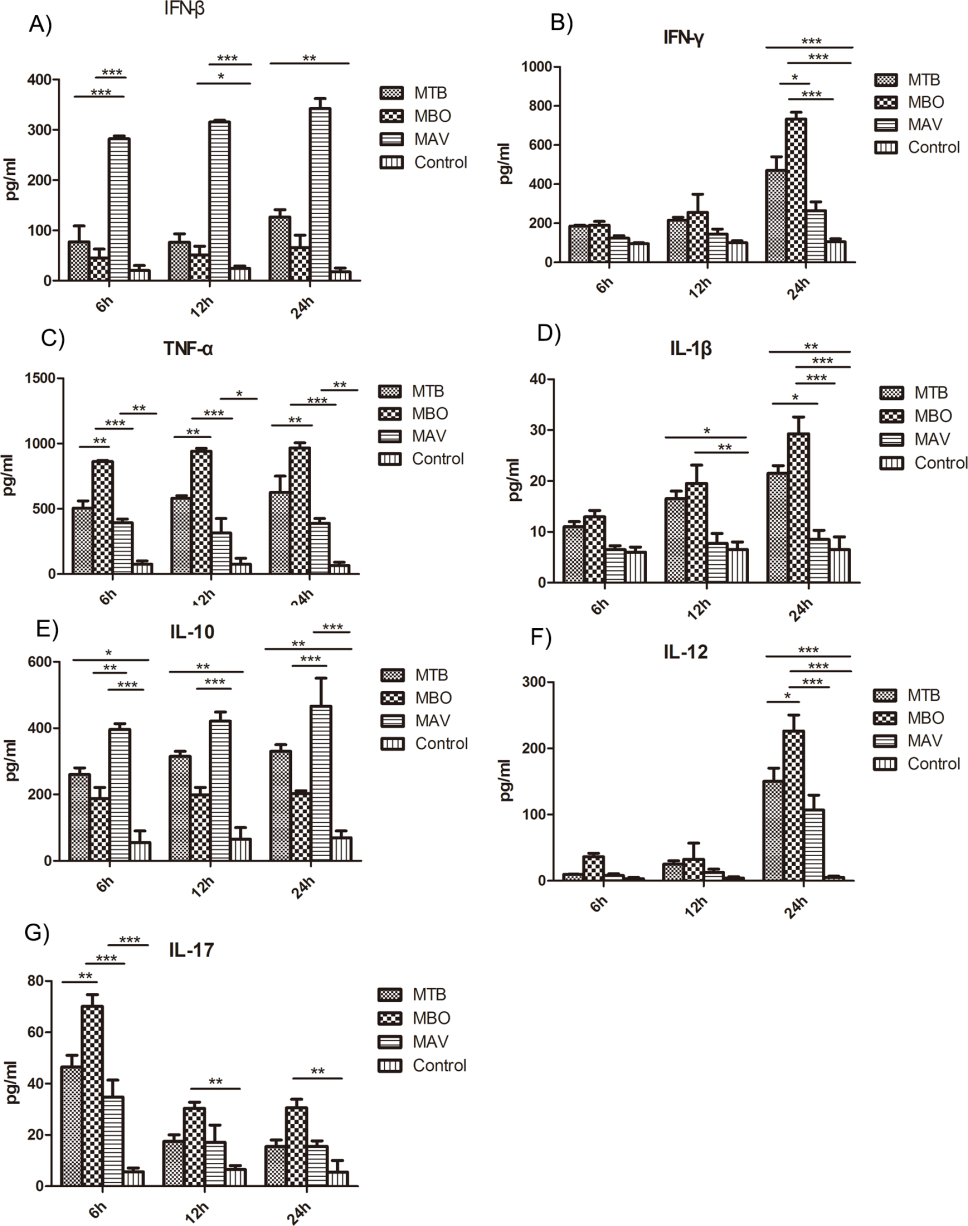
Expression of cytokines from cells infected with different bacterial isolates. RAW264.7 were infected with different bacterial isolates for 10 MOI and the cell culture supernatants were collected at 6h, 12h, 24h post-infection. Cytokines shown are IFN-β (A), IFN-γ (B) and TNF-α(C). Interleukins related to the Th1, Th2 and Th17 immune pathways such as IL-1β (D), IL-10 (E), IL-12 (F) and IL-17 (G) were analyzed. Results from each group was averaged with the individual results from three experiments and shown as mean±SD. Statistical analysis was performed using one-way ANOVA. ***P<0.0001 were considered significantly different.

To determine the immune response after infection with different isolates, we measured cytokine expression related to the Th1, Th2, and immune pathways in vitro (Fig 8). IFN-β levels were significantly higher after infection compared to control (Fig 8A). However, cytokines levels were inversely correlated with the virulence of the isolates. IFN-β was less expressed in the MBO strain group than in the MTB and MAV strain groups. IFN-γ (Fig 8B), TNF-α (Fig 8C) and IL-12 (Fig 8F) are Th1 related cytokines. These cytokines had a higher expression in the MBO strain group than in the MTB group and were least expressed in the MAV strain group. In contrast, IL-10 (Fig 8E) levels were lower in the MBO group and higher in the MTB and MAV groups, which indicate that the Th2 immune response was stronger in the MAV and MTB groups. The Th17 related cytokine IL-17 (Fig 8G) was an early trigger of the inflammatory response. Their levels were higher in the MBO group than in the MTB and MAV groups. In addition, their levels were higher at 6hpi than at 12 or 24hpi. Expression levels of IL-1β, a mediator of inflammation, were significantly higher in the MBO group than in the MAV group (Fig 8D).

## Discussion

In this study, we comparatively evaluated the virulence of three strains of *mycobacterium* isolated from domestic animals or wildlife in an experimental mouse intranasal inhalation challenge model. Marked diversity among the three strains was observed in terms of severity of clinical signs, lung pathology, bacterial burden, and circulating cytokine responses. Only the MBO strain displayed lethality in mice when administered in high doses. When we compare the pathogenicity of these strains of tuberculosis with that of the H37Rv strain in mice [9, 10], we find that MBO can induce more extensive lesions and rapid progression to death than H37Rv in mice infected with the same dose of bacteria. On the other hand, the MTB and MAV strains showed less pathogenicity than H37Rv. More than one hundred years ago the virulence of MTB and MBO was already reported as being different in human and bovine by Theobald Smith and Robert Koch [11, 12]. More recently, other studies have reinvestigated the virulence of MTB in cattle and other animals with novel technologies at higher resolution [13]. Cattle infected with MTB and MBO and sacrificed 17 weeks post-infection showed strong immune responses triggered by both organisms. However the MTB infected animals showed no pathology and less bacterial burden compared with the MBO infected animals [13]. We can conclude that the MTB strain can infect ruminants, showing less virulence than *M*. *bovis*.

In this research, the MTB strain was isolated from beef cattle and caused less pathological damage than the MBO strain. While reports of MBO infection in humans are rare, the MTB strain has been isolated from animals where animal-human contact is frequent and intimate, suggesting that interspecies transmission is possible [14]. Researchers have found that herd prevalence of comparative intradermal tuberculin (CIDT) reactors was 9.4% and was higher in herds owned by households with TB than in herds owned by TB free households in Ethiopia [15]. On the other hand, Srivastava et al found MTB in milk from cattle with MTB infection, which indicates the potential threat of transmission from cattle back to humans [16].

The genetic and molecular basis that leads to different virulence between MTB and MBO have been studied for many years. A factor called mycoside B, a monosaccharide phenolic glycolipid (PGL), which is expressed in MBO but not in MTB has been proposed as an explanation for its enhanced pathogenicity [17, 18]. Years later, Clifton E. Barry III et al found that in hypervirulent MTB, disruption of PGL synthesis would lead to complete loss of virulence. On the other hand, over expression of PGL in MTB or addition of the purified PGL in monocyte-derived macrophages can increase their virulence and innate immune responses [19]. MPB70 and MPB83 are homologous cross-reactive secreted mycobacterial proteins that play a role in delayed-type hypersensitivity response [20]. They are usually used as a capture antigen in the diagnosis of bovine tuberculosis [21, 22]. With *mycobacterium*, it has been reported that MPB70 and MPB83 are expressed at higher levels in MBO than in MTB [23]. Furthermore, it is known that sigma factor K (SigK) can positively regulate the expression of MPB70 and MPB83 [24] and that MBO has a defect of Rv0444c (SigK repressor) or anti-SigK. Further support of this regulation comes from the finding that the N-terminal region of Rv0444c-encoded protein interacting with SigK was missing [24]. The PhoPR located in the DNA-binding domain can regulate transcription [25]. However, in contrast to MBO, there is a mutation in MTB which leads to the downregulation of PhoPR, with the loss of biologically active lipids [26] and reduced expression of the 6-kDa early antigenic target (ESAT-6) [27], likely leading to decreased virulence [28]. Experiments with mutations between different strains of MTB found that H37Ra is deficient in the expression of PhoPR compared to H37Rv, likely explaining its attenuation [25].

The function of IFN-β in antibacterial activity has been studied for a long time but it’s still unclear. IFN-β has been reported to play a major role in activating the inflammasome responses [29]. Avirulent mycobacteria such as *M*. *smegmatis* can generate AIM2-inflammasome activation by inducing the strong production of IFN-β. However *Mycobacterium tuberculosis* but not ESX-1 deficient mutant inhibits the AIM2-inflammasome activation and IFN-β production [30]. In contrast, other studies report that *Mycobacterium tuberculosis* can trigger the expression of IFN-β, which has the opposite effect suppressing activation of the NLRP3 inflammasome [31]. Other study found that a di-adenylate cyclase (disAor dacA) over-expressing *M*. *tuberculosis* strain can activate the interferon regulatory factor (IRF) pathway with enhanced levels of IFN-β, which can increase the autophagy in macrophages [32]. In our study, we observed that the levels of expression of IFN-β inversely correlated with bacterial virulence. In order to clarify the distinct immune responses triggered by different mycobacteria strains with their varied virulence, we measured cytokines related to the M1, M2, Th1, Th2 and Th17 cellular responses. From our results, we can see that virulent *M*.*bovis* induced higher levels of pro-inflammatory cytokines, such as TNF-α, IL-12 or IL-1β, than MTB and *M*.*avium*, which may cause severe pathology, lung edema and death in mice. Mcnab et al found that type I IFN can inhibit the expression of host-protective cytokines such as TNF-α, IL-12, and IL-1β and induce IL-10 [33]. This was also demonstrated in our results, showing that IFN-β levels increased after infection, being lowest for the MBO strain and highest for the *M*.*avium* strain. Less inhibition of IFN-β may contribute to the strongest Th1 cellular immune response in the MBO infection and the weakest response in *M*.*avium* infection. Due to the relative exacerbation of pro-inflammation cytokines, lung inflammation increased in the groups treated with virulent MBO in a dosage-dependent manner. On the other hand, the induction of IL-10, which is related to the immune suppression response, was lower.

Macrophages are very plastic cells in the haematopoietic system; they play a big role in homeostasis, development, tissue repair and immunity [34]. Macrophages can present three different phenotypes when they are activated by different cytokines. Macrophage classical activation (M1 phenotype) is beneficial to the Th1 immune response while macrophage acquired deactivation (M2 phenotype) is beneficial to the Th2 immune response. Levels of the M1 phenotype marker IFN-γ were the highest and those of the M2 phenotype marker IL-10 were the lowest for the virulent MBO strain, compared to those of the MTB and *M*.*avim* strains, demonstrating that the virulent MBO strain lead simultaneously to more cytotoxic but not cytoprotective effects than the other two mycobacterium.

TNF-α has been demonstrated to be the best anti-mycobacterial immunological factor in humans [35], it can control tuberculosis by promoting macrophage phagolysosomal fusion and apoptosis [36]. In our research, the level of TNF-α triggered by mycobacterium infection increased with increasing virulence of mycobacterium strains. This was seen with a worsening of the pathological observations of the lungs, with distinct reduction of free alveolar space concurrent with high inflammatory cell infiltration and edema at 22 days post-infection. Recent experiments have shown that the use of a TNF-α neutralizing antibody in mice exacerbated the lung inflammation in the early stages of MTB infection [37]. Di Paolo et al found that TNF-α failed to control MTB replication without the existence of IL-1 receptor and that the two cytokine signaling pathways cooperate to establish an optimal MTB control [38].

*Mycobacterium avium* is a nontuberculous mycobacteria which can be found widely in the environment in water, soil, dust [39] and in animals like cow, deer, sheep [40, 41] and humans [39]. Like *Mycobacterium tuberculosis*, *Mycobacterium avium* can invade host macrophages and avoid being killed by preventing phagosomal maturation [42], causing granuloma and chronic infection as a result. *Mycobacterium avium* is an opportunistic pathogen causing most of the underlying conditions, a likely reason for the lower severity of pathology caused by MAV in comparison to the other two bacteria.

In conclusion, using a mouse model, we confirmed differences in virulence, in a dose dependent manner, among the three strains of *mycobacteria* responsible for lesions in cattle or deer. This variation in virulence will need to be confirmed in a natural host model to delineate mechanisms of infectivity and transmission, to aid in the development of optimal diagnostics and mitigation strategies.

## Supporting information

S1 Fig The species of the three strains of *Mycobacterium* was confirmed by spoligotyping; the result was compared with H37Rv and BCG. (TIF)

S2 Fig Comparison of body weight, lung weight and bacterial burden with the same dosage after infection with different bacterial isolates. (TIF)
