# Supplementary figures and images for: Differences in pathogenicity of three animal isolates of Mycobacterium species in a mouse model
Source: PLoS One. 2017 Aug 24;12(8):e0183666. doi: 10.1371/journal.pone.0183666 (PMC5570376; doi:10.1371/journal.pone.0183666)

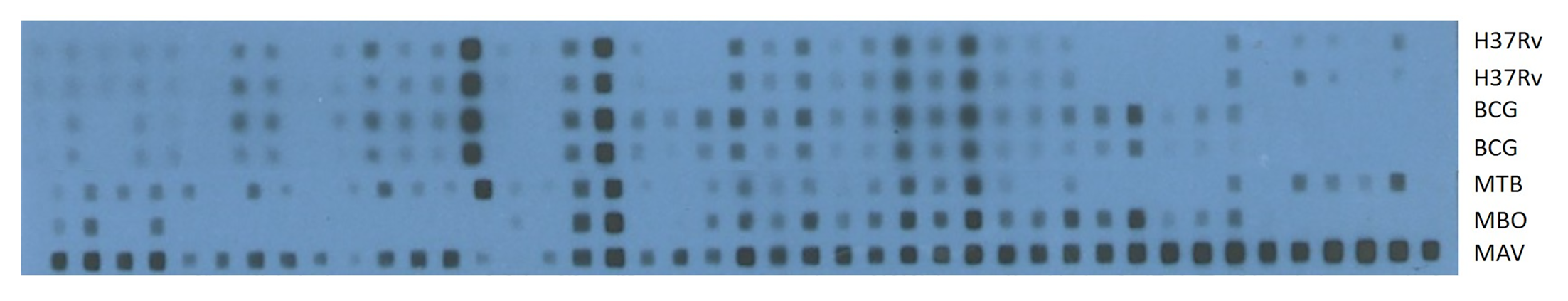

Supplement: S1 Fig — (TIF) [file pone.0183666.s001.tif]

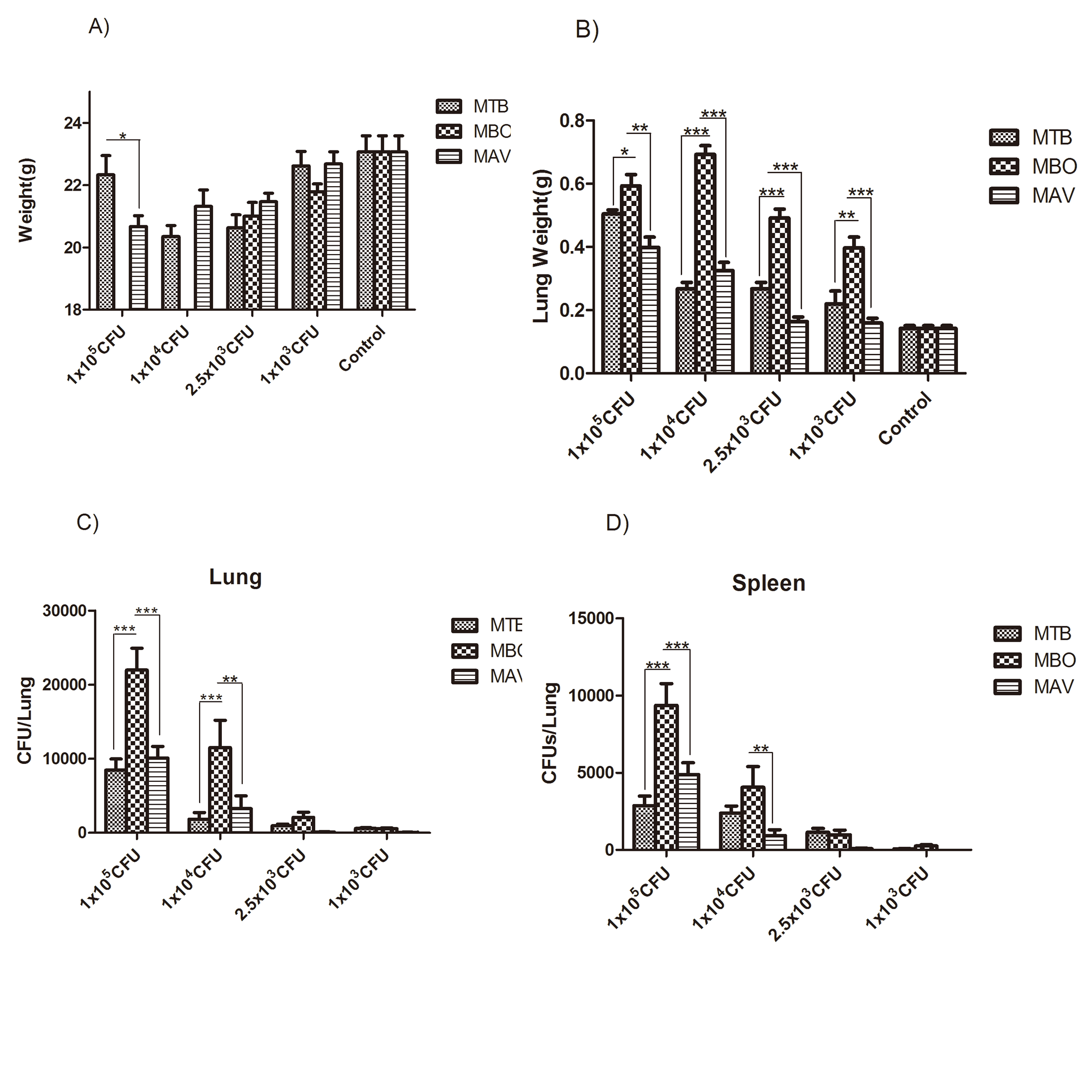

Supplement: S2 Fig — (TIF) [file pone.0183666.s002.tif]
